# *P*. *falciparum* infection and maternofetal antibody transfer in malaria-endemic settings of varying transmission

**DOI:** 10.1371/journal.pone.0186577

**Published:** 2017-10-13

**Authors:** Alistair R. D. McLean, Danielle Stanisic, Rose McGready, Kesinee Chotivanich, Caroline Clapham, Francesca Baiwog, Mupawjay Pimanpanarak, Peter Siba, Ivo Mueller, Christopher L. King, François Nosten, James G. Beeson, Stephen Rogerson, Julie A. Simpson, Freya J. I. Fowkes

**Affiliations:** 1 Burnet Institute, Melbourne, Victoria, Australia; 2 Centre for Epidemiology and Biostatistics, Melbourne School of Population and Global Health, The University of Melbourne, Melbourne, Australia; 3 Myanmar Oxford Clinical Research Unit, Yangon, Myanmar; 4 Institute for Glycomics, Griffith University, Gold Coast Campus, Southport, Queensland, Australia; 5 Shoklo Malaria Research Unit (SMRU), Mahidol-Oxford Tropical Medicine Research Unit, Faculty of Tropical Medicine, Mahidol University, Mae Sot, Thailand; 6 Centre for Tropical Medicine and Global Health, Nuffield Department of Medicine, University of Oxford, Oxford, United Kingdom; 7 Department of Clinical Tropical Medicine, Faculty of Tropical Medicine, Mahidol University, Bangkok, Thailand; 8 Papua New Guinea Institute of Medical Research, Madang, Papua New Guinea; 9 Population Health & Immunity Division, WEHI, Parkville, Victoria, Australia; 10 Malaria: Parasites & Hosts Unit, Institut Pasteur, Paris, France; 11 Department of Medical Biology, University of Melbourne, Parkville, Victoria, Australia; 12 Center for Global Health and Diseases, Case Western Reserve University, and Veterans Affairs Medical Center, Cleveland, OH, United States of America; 13 Department of Medicine, University of Melbourne, Melbourne, Australia; 14 Department of Microbiology and Central Clinical School, Monash University, Melbourne, Victoria, Australia; 15 Department of Epidemiology and Preventative Medicine, Monash University, Melbourne, Victoria, Australia; Universidade Federal de Minas Gerais, BRAZIL

## Abstract

**Introduction:**

During pregnancy, immunoglobulin G (IgG) is transferred from the mother to the fetus, providing protection from disease in early infancy. *Plasmodium falciparum* infections may reduce maternofetal antibody transfer efficiency, but mechanisms remain unclear.

**Methods:**

Mother-cord paired serum samples collected at delivery from Papua New Guinea (PNG) and the Thailand-Myanmar Border Area (TMBA) were tested for IgG1 and IgG3 to four *P*. *falciparum* antigens and measles antigen, as well as total serum IgG. Multivariable linear regression was conducted to assess the association of peripheral *P*. *falciparum* infection during pregnancy or placental *P*. *falciparum* infection assessed at delivery with maternofetal antibody transfer efficiency. Path analysis assessed the extent to which associations between *P*. *falciparum* infection and antibody transfer were mediated by gestational age at delivery or levels of maternal total serum IgG.

**Results:**

Maternofetal antibody transfer efficiency of IgG1 and IgG3 was lower in PNG compared to TMBA (mean difference in cord antibody levels (controlling for maternal antibody levels) ranged from -0.88 to 0.09, median of -0.20 log_2_ units). Placental *P*. *falciparum* infections were associated with substantially lower maternofetal antibody transfer efficiency in PNG primigravid women (mean difference in cord antibody levels (controlling for maternal antibody levels) ranged from -0.62 to -0.10, median of -0.36 log_2_ units), but not multigravid women. The lower antibody transfer efficiency amongst primigravid women with placental infection was only partially mediated by gestational age at delivery (proportion indirect effect ranged from 0% to 18%), whereas no mediation effects of maternal total serum IgG were observed.

**Discussion:**

Primigravid women may be at risk of impaired maternofetal antibody transport with placental *P*. *falciparum* infection. Direct effects of *P*. *falciparum* on the placenta, rather than earlier gestational age and elevated serum IgG, are likely responsible for the majority of the reduction in maternofetal antibody transfer efficiency with placental infection.

## Introduction

During pregnancy, immunoglobulin G (IgG) antibodies are transferred from mother to fetus. Maternally-derived antibodies provide the neonate with a pre-existing level of passive immunity to help protect from infection and disease. Vaccination of pregnant women can help protect infants against infectious diseases including influenza, tetanus and whooping cough [1]. These strategies rely on adequate maternofetal antibody transfer, but the factors that influence maternofetal antibody transfer are not fully understood. Clinical malaria caused by *Plasmodium falciparum* infection is uncommon during the first six months of life and, when *P*. *falciparum* infections are detected, they tend to be asymptomatic and are of lower parasite density than infections in older infants [2,3,4,5]. Protection against malaria during early infancy has been attributed to many factors [6] including the presence of maternal antibodies in the infant [7], though the relative contribution of maternal antibodies in mediating protection is unclear [8,9].

The humoral immune response is an important component of naturally acquired immunity to malaria and antibodies targeting blood-stage antigens (expressed by merozoites and infected erythrocytes) suppress high parasite densities and progression to symptomatic disease [10]. In malaria endemic areas, individuals develop naturally acquired immunity to *P*. *falciparum* infections with age, following repeated infections [10]. Breadth, magnitude and quality of antibody responses are critical, with antibody responses to a broad repertoire of antigenic targets associated with protection against symptomatic malaria in children [11,12,13,14]. Women who would otherwise be relatively immune to clinical *P*. *falciparum* malaria are again susceptible to malaria during pregnancy due in part to changes in immune function as well as the appearance of the placenta, a site of sequestration for *P*. *falciparum* variants that express *Pf*VAR2CSA on the erythrocyte surface [15]. The predominant antibody isotypes produced in response to malarial antigens are IgG1 and IgG3 [12,16,17,18], which are known to mediate complement deposition and opsonic phagocytosis, mechanisms that have been linked with protective immunity [19,20,21].

Several factors are known or suspected to influence the rate of maternofetal antibody transfer including total maternal IgG [22,23,24,25], gestational age at delivery [25,26,27,28,29], IgG subclass composition [28,30], HIV infection [22,29,31,32] and *P*. *falciparum* infection [22,23,25,31,33,34]. Infections with *P*. *falciparum* during pregnancy have been associated with earlier gestational age at delivery and increased maternal sera total IgG in some populations [23,34,35]; both preterm birth and increased total maternal IgG have been associated with reduced transport efficiency [22,23,24,26,27,28,30]. Placental *P*. *falciparum* infection may also impact maternofetal antibody transfer efficiency directly through the induction of pathological changes in the placenta including inflammation, deposition of pigment in fibrin or inflammatory cells, syncytial knotting and thickening of the trophoblastic basement membrane [36,37,38,39,40].

Studies investigating the relationship between placental *P*. *falciparum* infection and maternofetal antibody transfer efficiency of IgG (specific for tetanus [22,32,33], measles [22,29,34], respiratory syncytial virus [23,24] and malaria [25] antigens) have reported mixed results. Of the nine studies that have investigated associations between placental *P*. *falciparum* infection and maternofetal antibody transfer, three have found placental infection is associated with reduced antibody transfer efficiency for all antibodies investigated [32,33,34], four have noted reductions in transfer efficiency for some antibodies but not others [22,23,25,31], while two have reported no association [24,29]. Only one study has investigated the effect of *P*. *falciparum* infection on malaria-specific IgG, IgG1 and IgG3, reporting significantly lower maternofetal antibody transfer efficiency in women with any detectable *P*. *falciparum* infection at delivery of antibodies specific for some antigens, but not others [31]. Discrepancies in the above findings may be due to differences in antibodies studied, definitions of *P*. *falciparum* infection, transmission intensity, antimalarial treatment, location and statistical approaches.

To date, no study has directly reported associations between maternal *P*. *falciparum* infections detected in the periphery and antibody transfer efficiency. The relative contribution of gestational age at delivery and maternal sera IgG to associations between *P*. *falciparum* infection and reduced maternofetal transfer efficiency are yet to be elucidated. To address these gaps in the literature we sought to assess the effect of maternal *P*. *falciparum* infection during pregnancy and placental *P*. *falciparum* infection on maternofetal transfer efficiency of IgG1 and IgG3 against several malaria antigens and a non-malaria antigen (measles) in an area of high *P*. *falciparum* endemicity in Papua New Guinea (PNG), and an area of low *P*. *falciparum* endemicity at the Thailand-Myanmar Border Area (TMBA). We also investigated the extent to which any associations between *P*. *falciparum* infection and maternofetal transfer efficiency are explained through associations between *P*. *falciparum* infection and early gestational age at delivery or increased maternal sera IgG.

## Materials and methods

### Study population–Alexishafen, Papua New Guinea

Women attending prenatal care at Alexishafen Health Centre, Madang Province, Papua New Guinea (PNG) between August 2005 and September 2007 were recruited into a longitudinal study of malaria and pregnancy following voluntary informed consent [41]. Peripheral blood (5ml) was obtained from women at enrolment, delivery and any additional antenatal clinic visits. At delivery a placental biopsy and 10ml of cord blood were collected. Clinical and demographic data were obtained at enrolment and delivery visits. Presence of microscopic parasitaemia was determined from blood smears by two independent microscopists. A subset of 204 participants with available delivery and cord samples were included in the present study. The study was approved by The Medical Research Advisory Council of Papua New Guinea (MRAC 05/05) and the Human Research Ethics Committee of Melbourne Health, Australia (06/06).

### Study population—Shoklo Malaria Research Unit, Thailand-Myanmar Border Area

Women attending antenatal clinics of the Shoklo Malaria Research Unit, Thailand-Myanmar Border Area (TMBA), between November 1998 and January 2000 were invited to participate in a chloroquine prophylaxis randomized controlled trial for prevention of *P*. *vivax* [42]. *P*. *falciparum* episodes were similar in the chloroquine prophylaxis and placebo groups [42]. A total of 118 peripheral maternal blood and paired cord blood samples were utilised from a subset of pregnant women at delivery included in a previously reported study of malaria immunity [43]. At the Shoklo Malaria Research Unit, women are invited to attend an ANC as soon as they become aware of their pregnancy and were then encouraged to attend weekly thereafter.

### Method of gestational age assessment

Gestational age was estimated from Ballard scores [44] in PNG, and in TMBA either the Dubowitz method [45] or a calculation based on fundal height [46], which performed well compared to ultrasound in term newborns [47]. Studies conducted in resource-limited settings suggest that Ballard and Dubowitz methods give comparable estimates of gestational age [48,49], however there was no attempt to quality control gestational age assessment between sites. Ballard scores and the Dubowitz method both fall short of estimation of gestational age by early ultrasound assessment [50].

### *P*. *falciparum* exposures

In PNG, maternal *P*. *falciparum* peripheral infection during pregnancy was defined as any light microscopy detected infection in peripheral blood at any point during pregnancy. Women with *P*. *falciparum* infections detected were treated with curative doses of chloroquine and sulphadoxine pyrimethamine and all women were given unsupervised prophylaxis [41].

In TMBA, maternal *P*. *falciparum* peripheral infection during pregnancy was defined as any light microscopy detected infection in peripheral blood at any point during pregnancy. All women who attend TMBA ANCs were screened weekly for the presence of *P*. *falciparum* by light microscopy. When infections were detected they were treated immediately. For *P*. *falciparum* or mixed infections, a first infection was treated with quinine or artesunate and all women were given weekly chloroquine prophylaxis [42].

### Placental histology

In PNG only, placental histology was performed as described by Rogerson et al [37]. We compared the presence of any parasitaemia in the placenta (pathology class 1–3) to no parasites (pathology class 4–5). As a secondary analysis we compared the presence of parasites and monocytes with malaria pigment in the placenta (pathology class 2) to parasites without malaria pigment-containing monocytes (pathology class 1 and 3) and to no parasites (pathology class 4–5).

### Antibody determination at delivery

We measured total serum IgG; IgG1 and IgG3 antibodies to measles antigen (MEV-007—PROSPECbio, Rehovot, Israel) and four *P*. *falciparum* antigens (*Pf*EBA175_RII_ (3D7, amino acid position 146–713), *Pf*MSP2 (3D7, amino acid position 19–249), *Pf*AMA-1 (3D7, amino acid position 25–545) and *Pf*DBL5 (7G8, amino acid position 2003–2270)). We did not assess IgG2 or IgG4 levels, as these isotypes have previously been found to be present at very low levels against the *P*. *falciparum* antigens assessed [12,17,18], precluding a reliable assessment of maternofetal antibody transfer. Antibodies to these *P*. *falciparum* merozoite antigens are thought to be protective [51,52]. One of these *P*. *falciparum* antigens is known to induce IgG1 dominant responses (*Pf*AMA-1) and another is known to induce IgG3 dominant responses (*Pf*MSP2) [18,53]. Antibodies were measured using an enzyme-linked immunosorbent assay (ELISA) as described previously [43]. *P*. *falciparum* antigens were coated at 0.5 μg/ml, measles antigen at 1 μg/ml and goat anti-human-kappa at 1/1000. Horeseradish peroxidase (HRP)-conjugated goat anti-human IgG (Sigma), mouse anti-human IgG1 (Invitrogen) and mouse anti-human IgG3 (Invitrogen) were used as the secondary antibody (at 1/1000) in the total serum IgG, specific IgG1 and specific IgG3 assays respectively. The tertiary antibody (at 1/1000) for specific IgG1 and IgG3 assays was HRP-conjugated goat anti-mouse IgG (Merck Millipore). Assay output was related to a standard curve of pooled sera using a four-parameter logistic nonlinear regression model. TMBA samples had substantially lower levels of malaria-specific antibodies than PNG samples, so immunoassays against *P*. *falciparum* antigens were run at multiple concentrations of sera (sera dilutions are provided in S1 Table, range 1/100-1/20,000 for *P*. *falciparum* antigens). When assay reactivity from a sample was too high to reliably interpolate from the standard curve, then it was rerun at a lower sera concentration. The highest reactivity in an assay was set to 100 arbitrary units (AU). All wash steps were performed using PBS with 0.05% Tween and completed using an automated plate washer within a robotic platform (Perkin Elmer, Waltham, USA). Serum addition was performed using a robotic platform (Perkin Elmer, Waltham, USA). A sample was considered to be seropositive (or in the case of total IgG: elevated) if the AU for that sample was above the mean + 3 standard deviations of those for 15 non-exposed Melbourne blood donors.

### Statistical analysis

All statistical analyses were performed using Stata Version 13.1 (StataCorp, College Station, TX, USA). Correlations between cord and maternal antibodies were assessed using Spearman’s rank correlation coefficients. Wilcoxon rank-sum tests were used to assess differences in antibody levels between populations. Women who were seronegative for an antibody were not assessed for their ability to transfer that antibody. To assess the impact of study site on antibody transfer, multivariable linear regression of log_2_ cord antibody levels was performed with log_2_ maternal levels at delivery and study site as covariates, and further adjustment for the potential confounders, gravidity (primigravid/multigravid) and estimated gestational age at delivery (weeks). *P*. *vivax* infection was not included in the model.

To assess the impact of *P*. *falciparum* infection during pregnancy on cord antibody levels, all analyses were performed separately for each study site, as inherent differences in study design meant that the screening, treatment and recording of infections could not be considered equivalent for the two study sites. Multivariable linear regression was performed assessing the association of infection with log_2_ cord antibody levels after adjustment for log_2_ maternal antibody levels and gravidity (primigravid/multigravid). Effect modification by gravidity of the association between infection and cord antibody levels was assessed though the fitting of interaction terms between gravidity (primigravid/multigravid) and infection. Three different infection variables were used in the models: peripheral *P*. *falciparum* infection at any time during pregnancy (yes/no); placental histology (parasites/no parasites); placental histology (parasites and monocytes containing malaria pigment/parasites without monocytes containing malaria pigment/no parasites). Placental histology data were only recorded in the PNG study.

Where associations were observed between infection and maternofetal antibody transfer efficiency, path analysis was then conducted to investigate potential mediation of the relationship between infection and cord antibody levels through gestational age at delivery and maternal serum IgG. The proportion of the total effect due to an indirect association via gestation age at delivery or maternal serum IgG was then estimated.

## Results

### Study populations

IgG1 and IgG3 levels were determined for 204 maternal/cord pairs from Alexishafen, Papua New Guinea (PNG) and 118 pairs from the Thailand-Myanmar Border Area (TMBA). Gravidity and gestational age at delivery were higher in TMBA women relative to PNG women (Table 1). *P*. *falciparum* antibody seroprevalence, the proportion with detectable *P*. *falciparum* infections and the proportion with elevated serum IgG were lower in TMBA women relative to PNG women (Table 1).

**Table 1:** Distribution of maternal characteristics by study site.

	Alexishafen, Papua New Guinea (n = 204)	TMBA, Thailand (n = 118)
**Maternal characteristics**		
Age (years)	24 (21,28), [16–49]	25 (21–32), [15–42]
Gravidity	2 (1–4), [1–10]	3 (2–5), [1–13]
Gestational age at delivery (weeks)^a^	38 (37–40), [28–42]	40 (40,41), [31–42]
***Plasmodium* spp. infection**		
*P*. *falciparum* infection^b^	81 (40)	32 (27)
*P*. *vivax* infection^b^	12 (6)	32 (27)
*P*. *falciparum* placental histology		
No parasites detected	92 (45)	Not determined
Parasites detected	112 (55)	Not determined
**Antibody measures**		
Maternal seropositive		
*Pf*EBA175_RII_ IgG1	178 (87)	43 (36)
*Pf*EBA175_RII_ IgG3	199 (98)	114 (97)
*Pf*AMA1 IgG1	197 (97)	106 (90)
*Pf*AMA1 IgG3	189 (93)	91 (77)
*Pf*MSP2 IgG1	199 (98)	95 (81)
*Pf*MSP2 IgG3	204 (100)	106 (90)
*Pf*DBL5 IgG1	119 (58)	34 (29)
*Pf*DBL5 IgG3	123 (60)	74 (62)
Measles IgG1	195 (96)	113 (96)
Measles IgG3	204 (100)	117 (99)
Elevated serum IgG^c^	50 (25)	16 (14)

NB—Data presented as median (inter-quartile range), [minimum-maximum] or n (%). Abbreviations: TMBA = Thailand-Myanmar Border Area.

^a^ Estimated by Ballard scores in PNG, Dubowitz method in TMBA.

^b^ Infection detected by light microscopy (peripheral) at any point during pregnancy. 7 and 11 women experienced a *P*. *falciparum* infection and a *P*. *vivax* infection during pregnancy in Papua New Guinea and Thailand respectively.

^c^ Defined as a level of total IgG greater than the mean + three standard deviations of 15 Melbourne blood donors.

### Higher levels of malaria-specific IgG1 and IgG3 in maternal and cord sera from PNG than from TMBA

**Fig 1:**
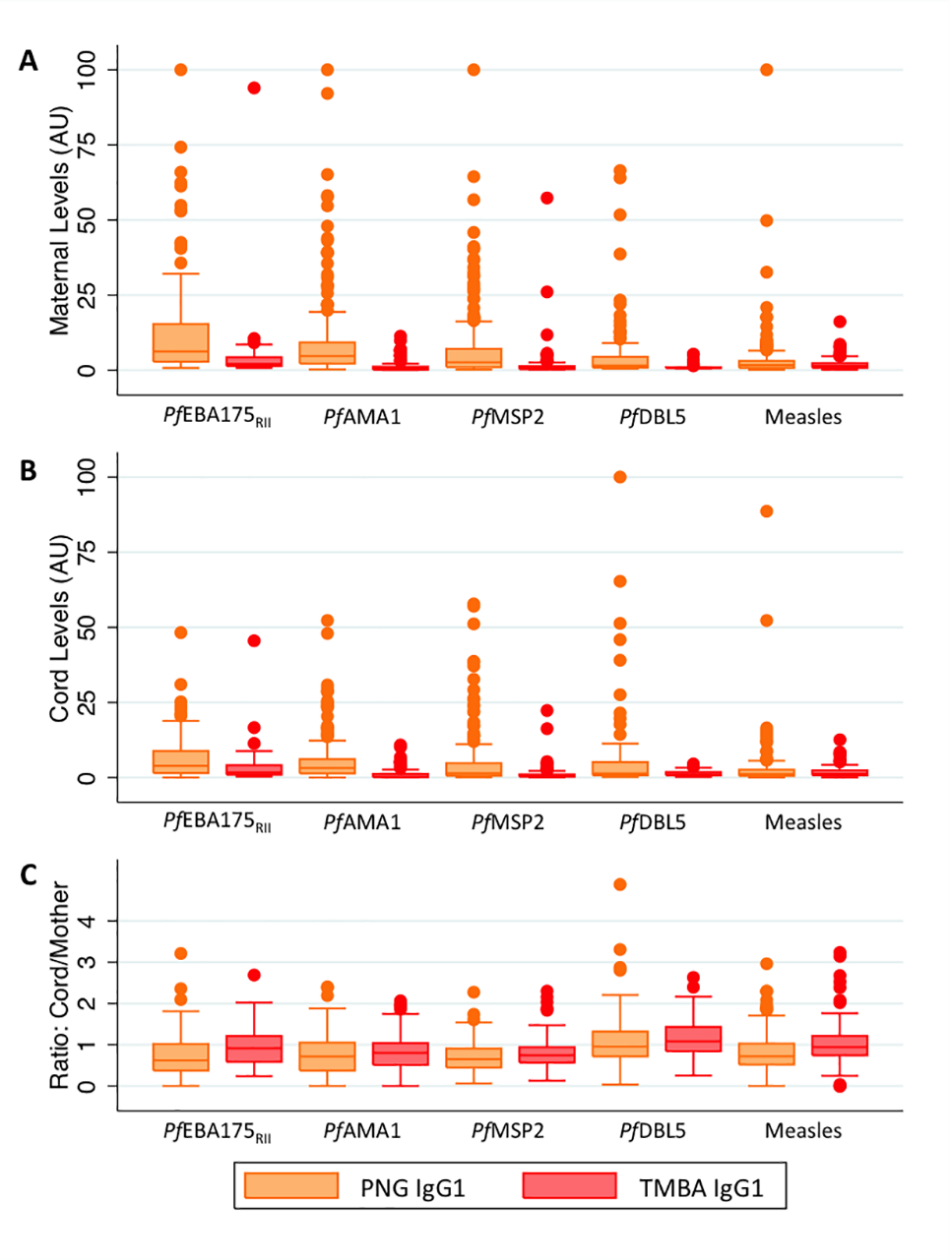
Maternal and cord IgG1 levels by study site. (A) Maternal IgG1 levels (arbitrary units) by study site. (B) Cord IgG1 levels (arbitrary units) by study site. (C) Cord:maternal IgG1 ratio by study site.

**Fig 2:**
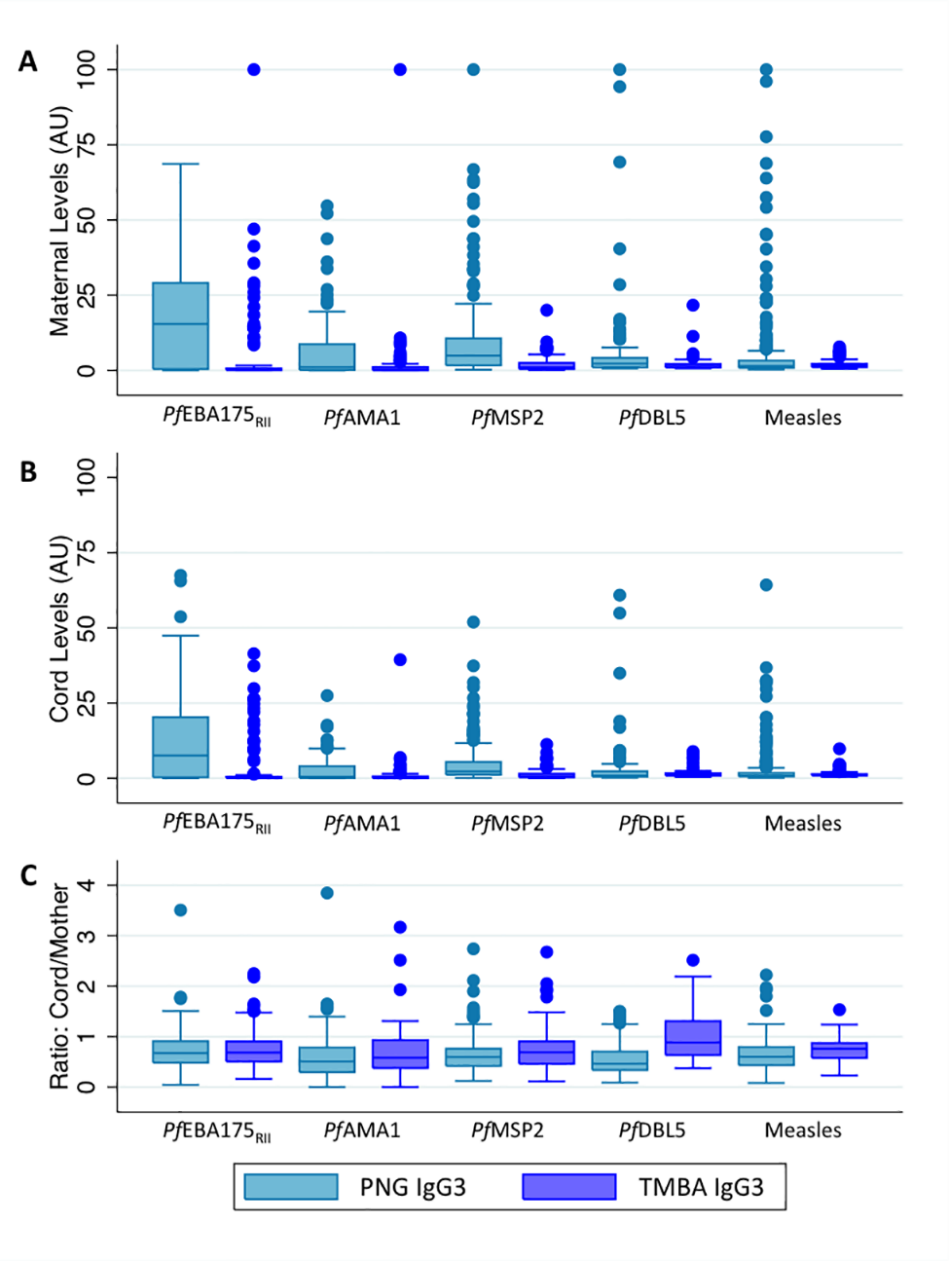
Maternal and cord IgG3 levels by study site. (A) Maternal IgG3 levels (arbitrary units) by study site. (B) Cord IgG3 levels (arbitrary units) by study site. (C) Cord:maternal IgG3 ratio by study site.

Maternal IgG1 and IgG3 levels were strongly correlated with cord IgG1 and IgG3 levels (Spearman’s ρ ranges from 0.78–0.96, S1 Fig). Maternal and cord IgG1 and IgG3 levels against *P*. *falciparum* antigens were higher in PNG samples, where malaria transmission is higher, than in TMBA samples (Figs 1 and 2, p<0.0001 for all comparisons except *Pf*DBL5 IgG3 (p = 0.16 and p = 0.58 for maternal and cord levels respectively)). IgG1 and IgG3 levels were lower in the cord blood than in the maternal blood for the majority of antigens (Figs 1C and 2C). The cord:maternal ratio of antibodies against all antigens (*P*. *falciparum* and measles) was lower in PNG samples than TMBA samples (Figs 1C and 2C). The cord:maternal IgG1 ratio was higher than the IgG3 ratio for each antigen investigated within each study site (Figs 1C and 2C).

### Maternofetal transfer efficiency of IgG1 and IgG3 antibodies specific for malaria and measles was lower in PNG women relative to TMBA women

To investigate maternofetal antibody transfer efficiency, linear regression was performed, modelling cord antibody levels (log_2_(AU)) with maternal levels (log_2_(AU)) included as a covariate. Maternofetal transfer efficiency of IgG1 and IgG3 antibodies specific for *P*. *falciparum* antigens was reduced in PNG compared to TMBA (Table 2) (estimate of adjusted mean difference of *P*. *falciparum* antibody levels in cord blood (log_2_ units) for PNG versus TMBA ranged from -0.88 to 0.09 with a median value of -0.15). The largest magnitude of effect was observed in DBL5 IgG3. Maternofetal transfer efficiency of antibodies specific for measles was significantly lower in PNG relative to TMBA (-0.41 log_2_ units (95% confidence interval (CI): -0.61, -0.21) and -0.26 (95% CI: -0.38, -0.14) for IgG1 and IgG3 respectively). Adjusting for gestational age at delivery and gravidity did not substantially alter the magnitude of associations observed (Table 2).

**Table 2:** Association of study site with maternofetal antibody transfer efficiency; estimated adjusted mean difference (95% confidence intervals) and p-values are presented.

	*Pf*EBA175_RII_−IgG1	*Pf*EBA175_RII_−IgG3	*Pf*AMA-1 –IgG1	*Pf*AMA-1 –IgG3	*Pf*MSP2 –IgG1	*Pf*MSP2 –IgG3	PfDBL5 –IgG1	PfDBL5 –IgG3	Measles–IgG1	Measles–IgG3
**Adjusted**^**1**^										
TMBA	Reference	Reference	Reference	Reference	Reference	Reference	Reference	Reference	Reference	Reference
PNG	-0.24 (-0.57,0.09); 0.16	-0.07 (-0.26,0.14); 0.53	-0.06 (-0.40,0.29); 0.75	-0.16 (-0.47,0.14); 0.29	-0.13 (-0.34,0.08); 0.22	0.09 (-0.12,0.29); 0.40	-0.28 (-0.64,0.07); 0.12	-0.88 (-1.11,-0.65); <0.001	-0.41 (-0.61,-0.21); <0.001	-0.26 (-0.39,-0.14); <0.001
**Adjusted**^**2**^										
TMBA	Reference	Reference	Reference	Reference	Reference	Reference	Reference	Reference	Reference	Reference
PNG	-0.11 (-0.45,0.23); 0.52	0.07 (-0.15,0.28); 0.53	0.10 (-0.25,0.45); 0.56	-0.03 (-0.35,0.29); 0.85	-0.04 (-0.26,0.19); 0.75	0.17 (-0.05,0.39); 0.14	-0.14 (-0.52,0.25); 0.48	-0.79 (-1.05,-0.53); <0.001	-0.34 (-0.56,-0.12); 0.002	-0.24 (-0.37,-0.11); 0.001

NB–Coefficients represent the differences in cord antibody levels by study site after adjustment for maternal antibody levels. Abbreviations: TMBA = Thailand-Myanmar Border Area; PNG = Papua New Guinea. 1 –Adjusted for log_2_ maternal levels. 2—Adjusted for log_2_ maternal levels, gravidity (primigravid/multigravid) and gestational age at delivery (weeks).

### Maternal *P*. *falciparum* infection, detected peripherally, was not associated with antibody transfer efficiency in either study site

**Fig 3:**
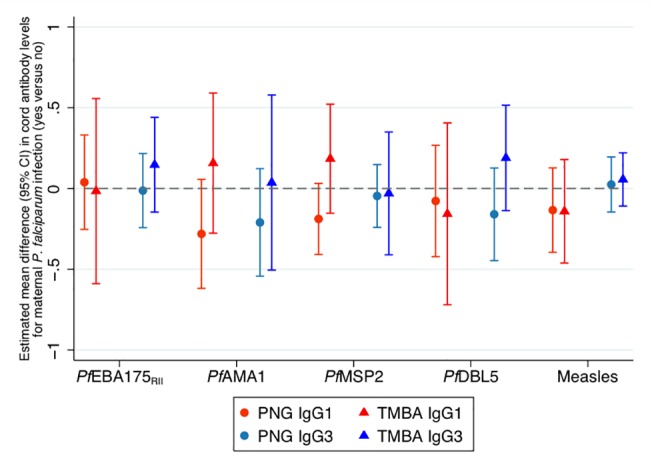
Association of maternal *P*. *falciparum* infection with maternofetal antibody transfer. Estimates and 95% confidence intervals are presented of the mean difference in log_2_ cord antibody levels after adjustment for log_2_ maternal antibody levels and gravidity, for mothers with a *P*. *falciparum* infection during pregnancy compared to uninfected mothers. Dashed line at 0 indicates no difference in mean log_2_ cord antibody levels. See S2 Table for a table version of this figure.

At PNG and TMBA there was no consistent association between peripheral *P*. *falciparum* infection and maternofetal IgG1 or IgG3 transfer efficiency (Fig 3, estimate of adjusted mean difference of antibody levels in cord blood (log_2_AU) for mothers with a peripheral *P*. *falciparum* infection versus those without a *P*. *falciparum* infection ranged from -0.15 to 0.21 with a median value of -0.05).

### Placental *P*. *falciparum* infection was associated with lower antibody transfer efficiency in primigravid women, but not multigravid women

**Fig 4:**
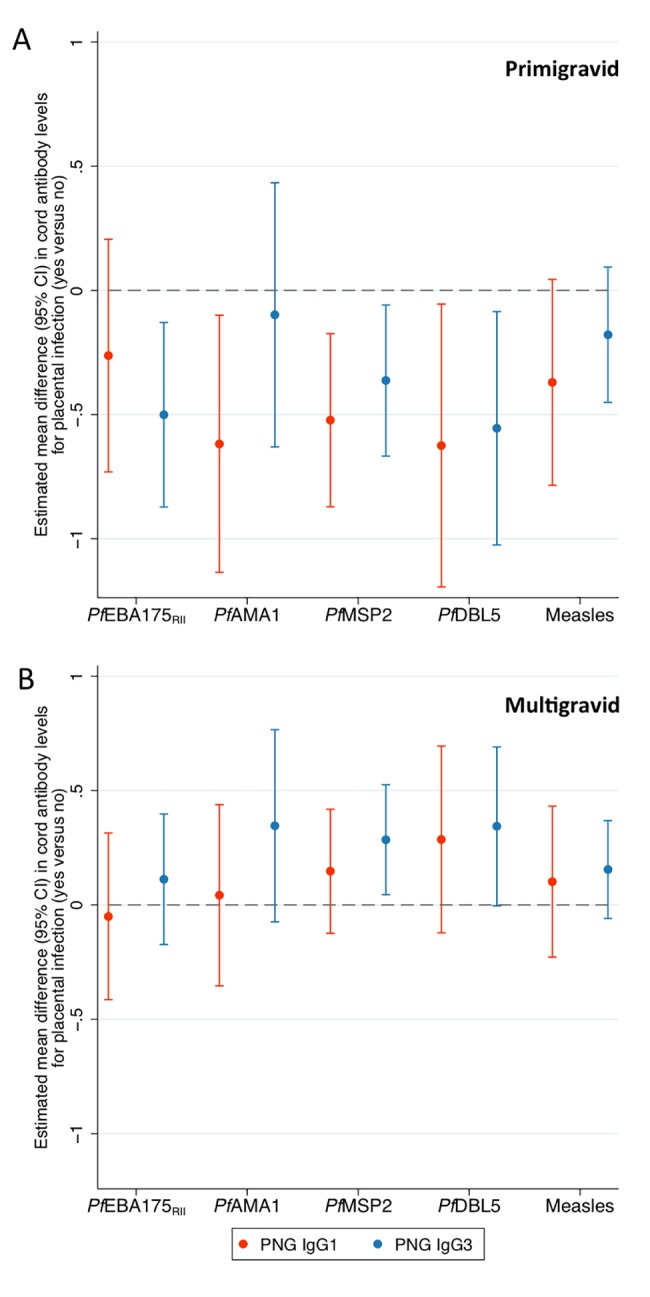
Association of placental infection with maternofetal antibody transfer efficiency in primigravid and multigravid Papua New Guinea women. Estimates and 95% confidence intervals are presented for the mean difference in log_2_ cord antibody levels after adjustment for log_2_ maternal antibody levels for **(A)** primigravid or **(B)** multigravid mothers with a *P*. *falciparum* placental infection compared to mothers with no placental infection. Dashed line at 0 indicates no difference in mean log_2_ cord antibody levels. See S3 Table for a table version of this figure, including estimates for all women.

No data were available on the placental histology of TMBA women, but a study conducted in the Shoklo and Maela camps during the time of this study indicated a low estimated prevalence of *P*. *falciparum* placental histopathological changes when early detection and treatment of infection, routine in this population, was performed [40]. Placental histology data were available from the PNG study allowing us to investigate the impact of placental infection on maternofetal antibody transfer efficiency. The presence of placental *P*. *falciparum* was associated with reduced maternofetal IgG1 and IgG3 transfer efficiency in primigravid women (Fig 4A, S3 Table) (estimate of mean difference of antibody levels in cord blood (log_2_ units) for mother-cord pairs with *P*. *falciparum* present in the placenta versus those without placental *P*. *falciparum* parasites present ranged from -0.62 to -0.10 with a median value of -0.36). There were no consistent associations between placental infection and maternofetal transfer efficiency in multigravid women (Fig 4B, S3 Table). In primigravid PNG women associations of placental infections with monocyte infiltrate were greater in magnitude than placental infections without monocyte infiltrate (S2 Fig).

### Placental *P*. *falciparum* infection was associated with lower antibody transfer efficiency in primigravid mothers primarily through mechanisms other than earlier gestational age at delivery or increased maternal IgG

**Fig 5:**
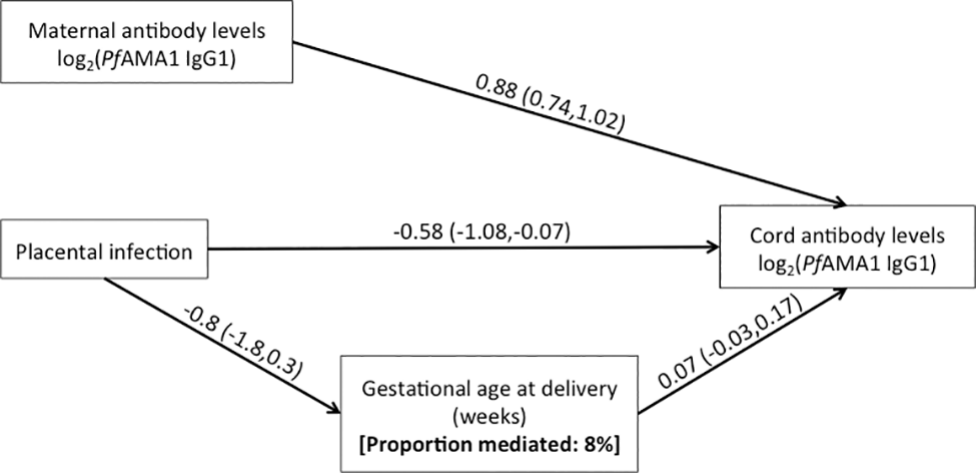
Path analysis model for cord PfAMA1 IgG1 levels and placental *P*. *falciparum* infection in primigravid women. Estimated mean difference (95% CI) are presented. For other antibodies see S4 Table.

Path analysis was performed to assess the proportion of the total effect of placental infection on maternofetal IgG1 and IgG3 transfer efficiency in PNG primigravid women mediated by gestational age and changes in total levels of maternal IgG. The negative effect of placental infection in primigravid women was not substantially mediated through induction of shorter gestational age at delivery (proportion of total effect attributable to gestational age mediation ranged from 0% to 18% with a median value of 5%, p ranged from 0.21 to 0.93 for indirect effects) (Fig 5, S4 Table). None of the effect appeared to be mediated through changes in total maternal serum IgG (p ranged from 0.63 to 0.87 for indirect effects).

## Discussion

To date, there has been considerable uncertainty regarding the effects of *P*. *falciparum* infection during pregnancy on maternofetal antibody transfer efficiency, as well as the mechanisms by which infection may mediate impaired maternofetal antibody transfer. A great strength of this study was the inclusion of women from two separate study sites with differing *P*. *falciparum* endemicity and access to antenatal clinics. Importantly, antibody data were standardised to a reference sera pool curve, which enabled direct comparisons of antibody levels across the study populations. The assessment of measles antibodies, which were present at similar levels in both study sites and should not be boosted during a placental *P*. *falciparum* infection, strengthened the generalisibility of the findings. Although some studies have included the potential mediating effects of elevated serum IgG and shorter gestation as confounders [22,23,31,33], this is the first study to investigate whether placental infection is mediated via these effects with path analysis. Malaria exposure during pregnancy was captured differently at each study site and therefore separate analyses had to be performed. The prevalence of HIV is very low (<1.5%) in these populations [54,55]) so HIV is unlikely to have a substantial effect on maternofetal transfer at the population level. The lack of placental histology data from the TMBA prevented an assessment of placental infection in that study site.

Maternofetal transfer efficiency of some antibodies was reduced in PNG compared to TMBA. Besides the direct effects of malaria, there are several factors that could account for this difference, including maternal genetic differences, gestational age at delivery and altered levels of total maternal IgG [22,23,32]. The median gestational age at delivery of PNG women was 2 weeks shorter than that observed at TMBA. However, differences in gestation cannot fully explain the difference we observed, given the persistence of an association between study site and maternofetal transfer efficiency of some antibodies even after controlling for gestational age at delivery. Very high levels of total maternal IgG are thought to saturate available receptors at the placenta, thereby reducing efficiency of transfer [56], however we did not observe an association between increased maternal IgG and decreased maternofetal transfer efficiency in either population in this study.

Greater maternofetal antibody transfer efficiency at TMBA relative to PNG may be due in part, to the prompt detection and treatment of *P*. *falciparum* infections at the TMBA site, preventing the persistence of long-term infections and thereby reducing the risk and consequences of placental infection. Women at TMBA were encouraged to attend antenatal clinics weekly, and were rapidly treated upon diagnosis, so the duration of any *Plasmodium* spp. infections during gestation is minimal and placental infections are rare [40]. In PNG, policy dictated weekly chloroquine prophylaxis, this was not monitored and so infections with *P*. *falciparum* may have persisted undetected. Malaria transmission was also substantially higher at the PNG site. At delivery, the majority of women presented with *P*. *falciparum* infected placentae in PNG, whereas at TMBA, placental infections are a rare outcome [40].

In primigravid PNG women we observed an association between the presence of *P*. *falciparum* parasites in the placenta and reduced maternofetal IgG1 and IgG3 transfer efficiency relative to uninfected women; no association was observed in multigravid PNG women. Primigravid women tend to have placental infections of greater parasite density and more commonly have associated placental inflammation than do multigravid women [57]. Multigravid women are not only to be less likely to present with placental infection, but also less likely to have impaired maternofetal antibody transfer when infected.

There are numerous mechanisms by which placental *P*. *falciparum* infection may reduce maternofetal antibody transfer efficiency. Placental *P*. *falciparum* infection is associated with a shorter gestational age [35] and shorter gestational age is associated with reduced maternofetal antibody transfer [26,27,28]. In our model, we found that gestational age explained a small proportion of the reduced maternofetal antibody transfer efficiency associated with placental infection. Placental infection has been associated with increased total maternal IgG in some other populations [23,34]; transfer efficiency can be reduced when maternal IgG levels are elevated [56]. However, amongst PNG women we did not observe a reduction in antibody transfer efficiency among those women with increased total serum IgG, as defined by levels greater than the mean plus three standard deviations of Melbourne control levels. The majority of the association between placental infection and reduced maternal transfer efficiency in primigravid PNG women was explained through mechanisms other than gestational age and maternal IgG in the present study. Our results indicate that a large proportion of the observed effect may be due to direct effects of placental infection. Given that placental infections with malaria-pigment containing monocytes tended to have even lower transfer efficiency than placental infections without monocytes, inflammation at the placenta is likely to be involved. Disruptions to the placental architecture that occur during placental infection [36,58,59,60] may have a negative impact on maternofetal transfer and receptor expression. Placental infection with inflammation seems to impair transplacental transport of glucose and amino acids via a reduction in expression of transport receptors [61,62]; if expression of placental immunoglobulin transport receptors were also reduced then this would explain some of the reduction in antibody transfer efficiency. As IgG1 outcompetes IgG3 for placental receptor binding [63], a reduction in transport receptors would likely have a greater impact on IgG1 transfer relative to IgG3 transfer. Further research is needed to elucidate the precise mechanisms by which placental infection with *P*. *falciparum* mediates reduced efficiency of maternofetal transfer, but our findings indicate direct effects of *P*. *falciparum* placental infection play a substantial role, emphasising the need for highly efficacious prevention in pregnancy.

Our study was subject to limitations. Our knowledge of infection status over the entire course of pregnancy was incomplete as the infection status of a woman was necessarily restricted to instances when they presented to antenatal care. Infection was detected via microscopy, so sub-microscopic infections were not captured. The infection status of cord blood was not determined in both study settings. Notably in the TMBA study we lacked placental histology data, limiting our analysis of placental histology exposures to pregnant women from PNG. We did not have data available on the outcomes of the children in their first year of life, precluding an assessment of the relationship between maternofetal antibody transfer and infant outcomes.

We have observed that placental *P*. *falciparum* infection was associated with reduced maternofetal antibody transfer efficiency in primigravid women and that only a small proportion of this association is mediated by gestational age. Adequate maternofetal transfer of antibodies is essential for maternal vaccination strategies to effectively protect infants; efforts to prevent and treat malaria in pregnancy should continue to be encouraged.

## Supporting information

S1 Table Assay sera dilutions. (DOCX)

S2 Table Effect of peripheral *P*. *falciparum* infection during pregnancy on log_2_ cord levels after adjustment for log_2_ maternal levels and gravidity; estimated adjusted mean difference (95% confidence intervals) and p-values are presented. (DOCX)

S3 Table Effect of placental infection on log_2_ cord levels after adjustment for log_2_ maternal levels in primigravid and multigravid women; estimated adjusted mean difference (95% confidence intervals) and p-values are presented. (DOCX)

S4 Table Proportion of total effect of placental infection mediated by gestational age at delivery in primigravid women. (DOCX)

S1 Fig Maternal and cord antibody levels are highly correlated. Scatter plots of cord and maternal IgG1 and IgG3 levels against *Pf*EBA175RII, *Pf*AMA1, *Pf*MSP2, *Pf*DBL5 and Measles. Samples from Alexishafen, Papua New Guinea are denoted as green closed circles and from Shoklo Malaria Research Unit, Thailand-Myanmar Border Area as closed red circles. Spearman ρ values (IgG1 and IgG3): *Pf*EBA175RII (0.91,0.96); *Pf*AMA1 (0.89,0.95); *Pf*MSP2 (0.94,0.94); *Pf*DBL5 (0.79,0.78); Measles (0.87,0.86).
(TIF)

S2 Fig Association of placental infection (with and without monocyte infiltrate) with maternofetal antibody transfer efficiency in primigravid and multigravid Alexishafen women. Estimates and 95% confidence intervals are presented for the mean difference in log_2_ cord antibody levels after adjustment for log_2_ maternal antibody levels for **(A)** primigravid or **(B)** multigravid mothers with a *P*. *falciparum* placental infection without monocyte infiltrate (orange triangles, n = 22 and n = 59 in primigravid and multigravida respectively) or a *P*. *falciparum* placental infection with monocyte infiltrate (blue triangles, n = 23 and n = 8 in primigravid and multigravida respectively) compared to mothers with placentas with no *P*. *falciparum* parasites present. Dashed line at y = 0 indicates no difference in mean log_2_ cord antibody levels.
(TIF)
