# Supplementary material for: P. falciparum infection and maternofetal antibody transfer in malaria-endemic settings of varying transmission
Source: PLoS One. 2017 Oct 13;12(10):e0186577. doi: 10.1371/journal.pone.0186577 (PMC5640245; doi:10.1371/journal.pone.0186577)
Supplement: S1 Table — (DOCX) [file pone.0186577.s001.docx]

**S1 Table. Assay sera dilutions**

| Assay | Sera dilution 1 | Sera dilution 2 | Sera dilution 3 |
| --- | --- | --- | --- |
| *Pf*DBL5^a^ – IgG1 | 1/100 | 1/800 |  |
| *Pf*DBL5^a^ – IgG3 | 1/100 | 1/800 |  |
| *Pf*MSP2^b^ - IgG1 | 1/300 | 1/1,200 |  |
| *Pf*MSP2^b^ - IgG3 | 1/1,000 | 1/4,000 | 1/10,000 |
| *Pf*AMA1^c^ - IgG1 | 1/250 | 1/2,000 | 1/16,000 |
| *Pf*AMA1^c^ - IgG3 | 1/250 | 1/1,000 | 1/4,000 |
| *Pf*EBA175_RII_^d^ - IgG1 | 1/2,000 | 1/8,000 | 1/20,000 |
| *Pf*EBA175_RII_^d^ – IgG3 | 1/1,000 | 1/4,000 | 1/10,000 |
| Measles^e^ – IgG1 | 1/200 |  |  |
| Measles^e^ – IgG3 | 1/200 |  |  |
| Total Sera IgG | 1/600,000 |  |  |

In all assays secondary (Ms anti-human IgG/IgG1/IgG3, Invitrogen) and tertiary (Goat anti-mouse HRP conjugated, Invitrogen) antibodies were diluted to 1/1000.

^a^Avril, M. et al. Immunization with VAR2CSA-DBL5 recombinant protein elicits broadly cross-reactive antibodies to placental Plasmodium falciparum-infected erythrocytes. Infect. Immun. 78, 2248–2256 (2010).

^b^Stanisic, D. I. et al. Immunoglobulin G subclass-specific responses against Plasmodium falciparum merozoite antigens are associated with control of parasitemia and protection from symptomatic illness. Infect. Immun. 77, 1165– 1174 (2009).

^c^Hodder, A. N., Crewther, P. E. & Anders, R. F. Specificity of the protective antibody response to apical membrane antigen 1. Infect. Immun. 69, 3286–3294 (2001).

^d^Richards, J. S. et al. Identification and prioritization of merozoite antigens as targets of protective human immunity to Plasmodium falciparum malaria for vaccine and biomarker development. J. Immunol. 191, 795–809 (2013).

^e^PROSPECbio
