# Supplementary material for: P. falciparum infection and maternofetal antibody transfer in malaria-endemic settings of varying transmission
Source: PLoS One. 2017 Oct 13;12(10):e0186577. doi: 10.1371/journal.pone.0186577 (PMC5640245; doi:10.1371/journal.pone.0186577)
Supplement: S2 Table — (DOCX) [file pone.0186577.s002.docx]

**S2 Table. Effect of peripheral *P. falciparum* infection during pregnancy on log_2_ cord levels after adjustment for log_2_ maternal levels and gravidity; estimated adjusted mean difference (95% confidence intervals) and p-values are presented.**

|  | *Pf*EBA175_RII_ – IgG1 | *Pf*EBA175_RII_ – IgG3 | *Pf*AMA-1 – IgG1 | *Pf*AMA-1 – IgG3 | *Pf*MSP2 – IgG1 | *Pf*MSP2 – IgG3 | PfDBL5 – IgG1 | PfDBL5 – IgG3 | Measles – IgG1 | Measles – IgG3 |
| --- | --- | --- | --- | --- | --- | --- | --- | --- | --- | --- |
| TMBA | -0.07  (-0.73,1.20); 0.82 | 0.12  (-0.18,0.43); 0.43 | 0.15  (-0.31,0.60); 0.52 | -0.04  (-0.62,0.55); 0.89 | 0.21  (-0.15,0.57); 0.25 | 0.16  (-0.26,0.57); 0.45 | -0.09  (-0.80,0.62); 0.79 | 0.21  (-0.14,0.56); 0.23 | -0.11  (-0.44,0.23); 0.53 | 0.06  (-0.12,0.23); 0.52 |
| PNG | 0.06  (-0.24,0.36); 0.69 | -0.05  (-0.29,0.18); 0.67 | -0.11  (-0.45,0.25); 0.55 | -0.12  (-0.46,0.22); 0.49 | -0.14  (-0.37,0.08); 0.21 | 0.00  (-0.20,0.20); 0.98 | 0.01  (-0.33,0.36); 0.94 | -0.05  (-0.35,0.23); 0.71 | -0.08  (-0.34,0.19); 0.57 | -0.05  (-0.22,0.13); 0.59 |
