# Supplementary material for: P. falciparum infection and maternofetal antibody transfer in malaria-endemic settings of varying transmission
Source: PLoS One. 2017 Oct 13;12(10):e0186577. doi: 10.1371/journal.pone.0186577 (PMC5640245; doi:10.1371/journal.pone.0186577)
Supplement: S3 Table — (DOCX) [file pone.0186577.s003.docx]

**S3 Table. Effect of placental infection on log_2_ cord levels after adjustment for log_2_ maternal levels in primigravid and multigravid women; estimated adjusted mean difference (95% confidence intervals) and p-values are presented.**

|  | *Pf*EBA175_RII_ – IgG1 | *Pf*EBA175_RII_ – IgG3 | *Pf*AMA-1 – IgG1 | *Pf*AMA-1 – IgG3 | *Pf*MSP2 – IgG1 | *Pf*MSP2 – IgG3 | PfDBL5 – IgG1 | PfDBL5 – IgG3 | Measles – IgG1 | Measles – IgG3 |
| --- | --- | --- | --- | --- | --- | --- | --- | --- | --- | --- |
| **All women** |  |  |  |  |  |  |  |  |  |  |
| No parasites | Reference | Reference | Reference | Reference | Reference | Reference | Reference | Reference | Reference | Reference |
| Placental parasites | -0.13  (-0.42,0.16); 0.38 | -0.11  (-0.35,0.12); 0.33 | -0.20  (-0.52,0.12); 0.22 | 0.18  (-0.16,0.51); 0.30 | -0.11  (-0.33,0.11); 0.35 | 0.04  (-0.16,0.23); 0.72 | -0.02  (-0.37,0.32); 0.90 | 0.03  (-0.27,0.32); 0.86 | -0.08  (-0.34,0.18); 0.54 | 0.03  (-0.14,0.20); 0.75 |
| **Primigravid** |  |  |  |  |  |  |  |  |  |  |
| No parasites | Reference | Reference | Reference | Reference | Reference | Reference | Reference | Reference | Reference | Reference |
| Placental parasites | -0.26  (-0.73,0.21); 0.27 | -0.50  (-0.87,-0.13); 0.01 | -0.62  (-1.14,-0.10); 0.02 | -0.10  (-0.63,0.44); 0.72 | -0.62  (-1.20,-0.05); 0.03 | -0.56  (-1.03,-0.08); 0.02 | -0.52  (-0.87,-0.17); 0.004 | -0.36  (-0.67,-0.06); 0.02 | -0.37  (-0.79,0.05); 0.08 | -0.18  (-0.45,0.10); 0.20 |
| **Multigravid** |  |  |  |  |  |  |  |  |  |  |
| No parasites | Reference | Reference | Reference | Reference | Reference | Reference | Reference | Reference | Reference | Reference |
| Placental parasites | -0.05  (-0.42,0.32); 0.79 | 0.11  (-0.18,0.40); 0.44 | 0.04  (-0.36,0.44); 0.84 | 0.35  (-0.08,0.77); 0.11 | 0.29  (-013,0.70); 0.17 | 0.34  (-0.01,0.69); 0.06 | 0.15  (-0.13,0.42); 0.29 | 0.28 (0.04,0.53); 0.02 | 0.10  (-0.23,0.43); 0.55 | 0.15  (-0.06,0.37); 0.16 |
| p value for interaction | 0.48 | 0.01 | 0.05 | 0.19 | 0.01 | 0.003 | 0.003 | 0.001 | 0.08 | 0.06 |
