# Supplementary material for: P. falciparum infection and maternofetal antibody transfer in malaria-endemic settings of varying transmission
Source: PLoS One. 2017 Oct 13;12(10):e0186577. doi: 10.1371/journal.pone.0186577 (PMC5640245; doi:10.1371/journal.pone.0186577)
Supplement: S4 Table — (DOCX) [file pone.0186577.s004.docx]

**S4 Table. Proportion of total effect of placental infection mediated by gestational age at delivery in primigravid women.**

|  | *Pf*EBA175_RII_  IgG1 | *Pf*EBA175_RII_  IgG3 | *Pf*AMA-1  IgG1 | *Pf*AMA-1  IgG3 | *Pf*MSP2  IgG1 | *Pf*MSP2  IgG3 | PfDBL5  IgG1 | PfDBL5  IgG3 | Measles  IgG1 | Measles  IgG3 |
| --- | --- | --- | --- | --- | --- | --- | --- | --- | --- | --- |
| Proportion effect attributable to weeks gestation | 8% | 15% | 8% | 9% | 1% | 2% | 2% | 0% | 2% | 18% |
| p value of indirect effect | 0.58 | 0.21 | 0.32 | 0.62 | 0.93 | 0.78 | 0.81 | 0.85 | 0.86 | 0.31 |
| p value of direct effect | 0.28 | 0.03 | 0.02 | 0.29 | 0.01 | 0.02 | 0.05 | 0.09 | 0.26 | 0.31 |
