# Supplementary figures and images for: P. falciparum infection and maternofetal antibody transfer in malaria-endemic settings of varying transmission
Source: PLoS One. 2017 Oct 13;12(10):e0186577. doi: 10.1371/journal.pone.0186577 (PMC5640245; doi:10.1371/journal.pone.0186577)

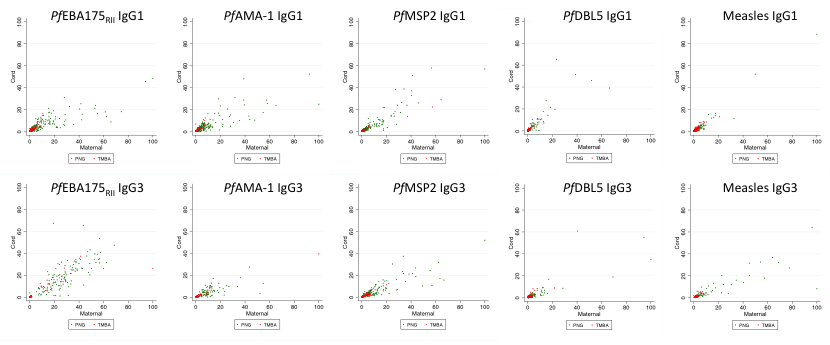

Supplement: S1 Fig — Scatter plots of cord and maternal IgG1 and IgG3 levels against PfEBA175RII, PfAMA1, PfMSP2, PfDBL5 and Measles. Samples from Alexishafen, Papua New Guinea are denoted as green closed circles and from Shoklo Malaria Research Unit, Thailand-Myanmar Border Area as closed red circles. Spearman ρ values (IgG1 and IgG3): PfEBA175RII (0.91,0.96); PfAMA1 (0.89,0.95); PfMSP2 (0.94,0.94); PfDBL5 (0.79,0.78); Measles (0.87,0.86). (TIF) [file pone.0186577.s005.tif]

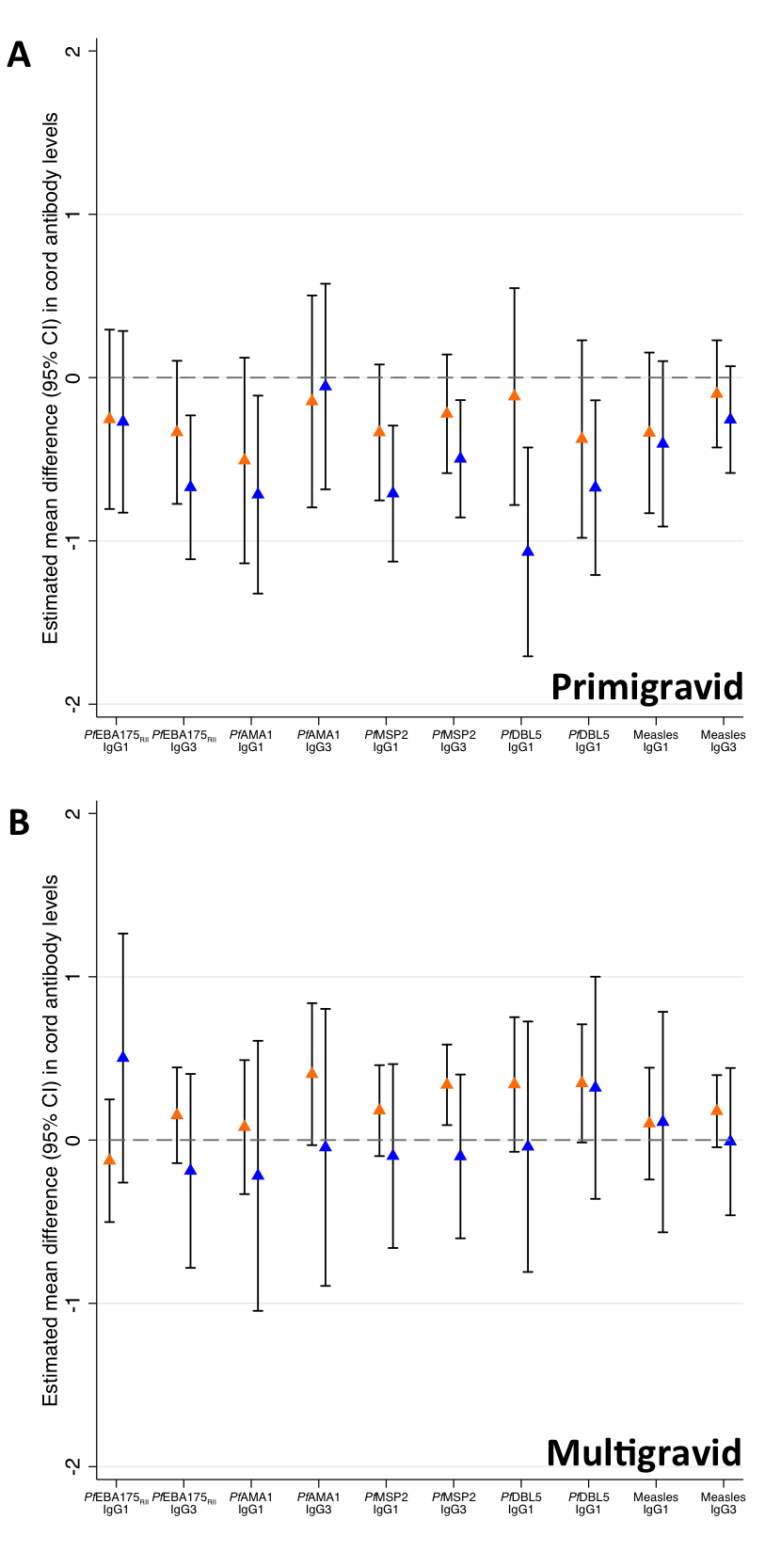

Supplement: S2 Fig — Estimates and 95% confidence intervals are presented for the mean difference in log2 cord antibody levels after adjustment for log2 maternal antibody levels for (A) primigravid or (B) multigravid mothers with a P. falciparum placental infection without monocyte infiltrate (orange triangles, n = 22 and n = 59 in primigravid and multigravida respectively) or a P. falciparum placental infection with monocyte infiltrate (blue triangles, n = 23 and n = 8 in primigravid and multigravida respectively) compared to mothers with placentas with no P. falciparum parasites present. Dashed line at y = 0 indicates no difference in mean log2 cord antibody levels. (TIF) [file pone.0186577.s006.tif]
